# Multimorbidity patterns and 15-year trajectories of physical performance: a population-based study

**DOI:** 10.1186/s12916-026-04828-6

**Published:** 2026-03-25

**Authors:** Francesco Palmese, Davide Liborio Vetrano, Caterina Gregorio, Amaia Calderón-Larrañaga, Anna-Karin Welmer, Alessandra Marengoni, Giorgio Bedogni, Marco Domenicali, Federico Triolo

**Affiliations:** 1 Aging Research Center, Department of Neurobiology, Care Sciences and Society, Karolinska Institutet and Stockholm University, Stockholm, Sweden; 2 Department of Medical and Surgical Sciences, Alma Mater Studiorum University of Bologna, Ravenna Campus, Ravenna, Italy; 3 Department of Primary Health Care, Internal Medicine Unit Addressed to Frailty and Aging, AUSL Romagna, Ravenna, Italy; 4 Stockholm Gerontology Research Center, Stockholm, Sweden; 5 Division of Physiotherapy, Department of Neurobiology, Care Sciences and Society, Karolinska Institutet, Stockholm, Sweden; 6 Women’s Health and Allied Health Professionals Theme, Medical Unit Medical Psychology, Karolinska University Hospital, Stockholm, Sweden; 7 Department of Clinical and Experimental Sciences, University of Brescia, Brescia, Italy

**Keywords:** Multimorbidity patterns, Physical performance, Functional decline, Walking speed, Chair stand test, Aging, Personalized medicine, Population-based study

## Abstract

**Background:**

Chronic diseases can impact physical function, yet little is known about how specific disease combinations relate to long-term physical performance trajectories and whether these associations vary across different performance measures. This population-based study explored the association between multimorbidity patterns and 15-year changes in physical performance among older adults.

**Methods:**

We analyzed 15-year longitudinal data on 3112 dementia-free individuals aged 60 and older participating in the Swedish National study on Aging and Care in Kungsholmen. Physical performance was assessed through walking speed and chair-stand tests, further combined into a z-standardized overall measure. Latent class analysis was used to identify groups of individuals with similar patterns of diseases. Linear mixed models were used to evaluate the association between multimorbidity patterns and changes in physical performance scores over time. Inverse probability weighting was used to account for attrition over the follow-up.

**Results:**

Four multimorbidity patterns were identified: 1) psychiatric, respiratory, & musculoskeletal, 2) anemia & sensory impairment, 3) cardiometabolic & inflammatory, and 4) unspecific. Compared to individuals without multimorbidity (≤ 1 disease), all patterns were associated with faster annual declines in physical performance, with the steepest decline observed for the cardiometabolic & inflammatory pattern (β*time = -0.066, 95%CI: -0.111, -0.021), followed by the anemia & sensory impairment pattern (β*time = -0.043, 95%CI: -0.063, -0.023). Results remained consistent after adjustment for the number of chronic diseases.

**Conclusions:**

Multimorbidity patterns are differentially associated with the rate of decline in physical performance, with the cardiometabolic & inflammatory pattern being associated with the fastest decrease. Classifying individuals according to multimorbidity patterns may help guide targeted strategies to preserve physical function in later life.

**Supplementary Information:**

The online version contains supplementary material available at 10.1186/s12916-026-04828-6.

## Background

As global populations age, preserving functional ability has become a core objective of public health and clinical care [1]. According to the World Health Organization (WHO), the term functional ability refers to the health-related attributes that enable older adults to do what they value most, resulting from the dynamic interplay between intrinsic capacity—comprising physical and mental function—and environmental factors [2]. Among these components, physical function is particularly valued by older individuals as it underpins independence, mobility, and the ability to participate in daily meaningful activities. Maintaining physical performance is thus a marker of healthy aging and a key target for prevention.

Physical function declines with age [3], but the rate and extent of this decline are highly variable [4, 5]. Objective measures of physical performance, such as walking speed and chair stand time, are sensitive tools for detecting early signs of functional deterioration [6, 7]. Walking speed has been shown to predict adverse health outcomes including hospitalization, disability, and mortality [8, 9], while the chair stand test reflects both strength and balance and is a reliable predictor of survival in older adults [10–13]. Given the modifiable nature of physical function, early identification of individuals at risk for physical decline is critical for delaying or preventing dependency and maintaining well-being in late life.

A major determinant of physical function in older adults is health status, especially the presence and progression of chronic diseases [14]. Conditions such as cardiovascular, musculoskeletal, and metabolic disorders adversely affect physical performance. This impact is amplified in the presence of multimorbidity, or multiple long-term conditions (MLTC), which is typically defined as the co-occurrence of two or more chronic diseases in the same individual [15, 16]. The prevalence of multimorbidity is rapidly increasing, largely driven by improvements in life expectancy and better disease detection and management [17]. Consequently, understanding how multimorbidity contributes to physical decline has become a public health priority.

Despite its growing recognition, much of the existing research has conceptualized multimorbidity using disease counts, which fails to capture the complexity and interactions among coexisting conditions. A complementary approach focuses on identifying data-driven multimorbidity patterns, i.e., distinct combinations of diseases that tend to co-occur more frequently than others in the population, based on shared risk factors or underlying pathophysiological mechanisms [1]. These patterns have shown differential prognostic implications across a range of health outcomes, including cognitive decline, frailty, disability, depression, and higher healthcare utilization [18–24]. Importantly, this approach may offer greater insight for risk stratification and targeted interventions than traditional count-based definitions [25].

However, only one study has investigated how data-driven multimorbidity patterns influence the long-term trajectories of objectively measured physical performance [26]. Thus, the aim of this study was to examine the association between distinct multimorbidity patterns and 15-year trajectories of physical performance in a community-based cohort of older adults.

## Methods

### Study design and study participants

Data were gathered from the Swedish National Study on Aging and Care in Kungsholmen (SNAC-K), an ongoing prospective study that involves adults aged 60 years or older, residing in the Kungsholmen district of Stockholm, Sweden. SNAC-K was approved by the Regional Ethical Review Board in Stockholm and the Swedish Ethical Review Authority, and all participants or next of kin (for cognitively impaired individuals) provided written informed consent. The baseline (2001–2004) of the study involved 3363 individuals from 11 age cohorts (60, 66, 72, 78, 81, 84, 87, 90, 93, 96, 99 + years), who were followed up every 6 years (< 78 years old) or 3 years (≥ 78 years old). The present study included data from the baseline wave to the fifth follow-up wave, spanning from 2001 to 2019. From the initial cohort, we excluded individuals with a clinical diagnosis of dementia at baseline (n = 241) to ensure that observed changes in physical performance reflected true physical decline rather than difficulties in completing performance-based tests due to severe cognitive impairment. The analytical sample therefore included 3112 individuals (Additional file 1: Figure S1 depicts the flow of participants, including lost to follow-up).

### Data collection

At each study wave, SNAC-K participants undergo a comprehensive clinical and functional assessment carried out by trained healthcare professionals. Consistent protocols are followed across all study waves to ensure uniform data collection. In cases of inability to attend the visit site, home visits are arranged.

### Physical performance assessment

Physical performance was assessed through walking speed [27] and the chair stand test [28]. Walking speed was timed as participants walked 6 m (m), or 2.4 m for those who considered themselves slow walkers [27]. A value of 0 s was assigned to participants unable to perform the test due to the following physical limitations: 1) not being able to stand up or walk without assistance; 2) using a wheelchair; 3) not being able to move around without assistance; 4) not being able to walk 100–200 m without great difficulty. For descriptive purposes, a previously defined cutoff of walking speed ≤ 0.8 m/s was used to identify slow walkers [29].

The chair stand was assessed as the time it took for the participant to stand up from a chair five times consecutively as quickly as possible [28]. The test was measured in seconds, with shorter time indicating greater lower limb muscle strength. A value of 52 s, the worst value recorded in our study population, was assigned to participants unable to perform the test due to physical limitations. For descriptive purposes, the cutoff of 15 s was used to identify impairment in the chair stand test [29].

Gait speed and chair stand were analyzed as separate outcomes, both in their original scale with the chair stand scale reversed for direction harmonization, and as z-scores based on baseline values to enable standardized comparisons of effect sizes. Furthermore, a global measure of physical performance was derived by averaging the z-scores of the two measures.

### Multimorbidity assessment

Chronic disease diagnoses relevant to older adults at baseline were identified using a previously developed algorithm. Specifically, 60 chronic disease categories were defined through a clinically guided grouping of 918 ICD-10 codes and used to derive data-driven multimorbidity patterns (see statistical analysis for details) [25]. Diagnoses were retrieved by physicians based on a comprehensive assessment that integrated data from physical examinations, medical histories, chart reviews, self-reports, and/or proxy interviews. Additional sources included clinical parameters, laboratory tests, medication records, and information from inpatient and outpatient national health registers. All conditions were coded according to the International Classification of Diseases, 10th Revision (ICD-10) [30].

### Vital status and loss to follow-up

Information about vital status during the follow-up period was derived from National Cause of Death Register by the Swedish National Board of Health and Welfare. Participants were considered lost to follow-up if they declined to participate, could not be contacted, had moved out of the study area, or cancelled an assessment.

### Other variables

For descriptive purposes, several baseline sociodemographic, lifestyle, and anthropometric characteristics were considered, including age, sex, education (elementary school, high school, and university or above), smoking status (never, former, or current smoker), alcohol consumption (nor or occasional; light to moderate, defined as 1–14 drinks per week for men or 1–7 drinks per week for women; or heavy, defined as ≥ 15 drinks per week for men or ≥ 8 drinks per week for women), and body mass index (BMI). The number of medications, coded in accordance with the Anatomical Therapeutic Chemical classification, was also reported at baseline. Lastly, global cognitive function was assessed at baseline using the Mini Mental State Examination (MMSE).

### Statistical analysis

Baseline characteristics were described using numbers and proportions for categorical variables and means with standard deviations (SD) or medians with interquartile ranges (IQR) for continuous variables, as appropriate. Differences in baseline characteristics across multimorbidity patterns were assessed using Chi-square tests for categorical variables, and Kruskal–Wallis rank tests for continuous variables.

Groups of individuals with similar diseases patterns at baseline were identified using latent class analysis (LCA). To minimize statistical noise, out of the 60 chronic diseases, only those with prevalence > 2% in the study population were considered in the analysis. The optimal number of latent classes was determined by comparing the adjusted BIC across models, with lower values indicating better model fit. Latent classes were labelled based on the chronic conditions that characterized them, defined by an observed-to-expected (O/E) ratio ≥ 2 and an exclusivity value (i.e., ratio between the number of disease cases in a class and the total number of individuals with that disease in the population) of ≥ 25%. Each participant was assigned to the class for which they had the highest posterior probability of membership.

Linear mixed models with random intercept and random slope were used to assess the association between baseline multimorbidity patterns and changes in physical performance over time, using follow-up time as the timescale. Baseline multimorbidity patterns served as the main exposures, with chronic conditions count groups evaluated as an alternative for comparison. In the primary analysis, the reference group consisted of individuals without multimorbidity. In a secondary analysis, restricted to individuals with multimorbidity (i.e., ≥ 2 chronic conditions), the Unspecific pattern was used as reference group. An interaction term between multimorbidity patterns and time (in years) was introduced to estimate the average annual decrease in physical function for a given pattern compared to the reference group. All the analyses were adjusted for baseline age, sex, education, BMI, MMSE, and number of medications, and were conducted using a complete case analysis (93% of the study cohort had complete data). To address potential bias due to attrition, inverse probability weights (IPW) were applied to account for censoring due to death or dropout during follow-up. IPW were derived by modelling the probability of death and drop-out given the individuals’ baseline characteristics, i.e., age, sex, education, BMI, MMSE, alcohol consumption, smoking status, disability status (computed as a score ranging from 0 to 14, derived from the sum of limitations in activities of daily living [ADL] and instrumental activities of daily living [IADL]), number of chronic diseases, and number of medications (see estimates without IPWs in Additional file 1: Table S10).

Three-way interactions between multimorbidity patterns, time, and sex and between multimorbidity patterns, time, and age were tested, and stratified analyses were conducted to explore potential effect modification. Sensitivity analyses were performed to assess the robustness of the findings after: (1) excluding individuals with less than two measures of physical performance tests during follow-up, (2) excluding individuals who developed dementia within the first 6 years of follow-up, (3) adjusting the linear mixed models for number of chronic diseases, (4) adjustment for institutionalization, and (5) assigning alternative imputed values to participants unable to perform the tests due to physical limitations, namely the 90th and 75th percentile values for walking speed (0.488 m/s and 0.813 m/s, respectively) and for the chair stands test (22 s and 16 s, respectively), given that test non-completion varied across waves and multimorbidity clusters (Additional file 1: Table S12 and Table S13).

Statistical analyses were carried out with STATA 18.0 (Stata Corp., College Station, TX, USA), and R, version 4.0.3, (R Foundation for Statistical Computing, Vienna, Austria) using the poLCA package for the LCA. In all analyses, a p-value < 0.05 was considered statistically significant.

## Results

### Multimorbidity pattern characterization

The analytical sample comprised 3112 dementia-free individuals, with median (IQR) age 72 (66;81) and 63.3% female participants. After applying latent class analysis to classify participants according to their chronic diseases, four multimorbidity patterns were detected: 1) Psychiatric, respiratory & musculoskeletal (*n* = 482), 2) Anemia & sensory impairment (*n* = 609), 3) Cardiometabolic & inflammatory (*n* = 354), and 4) Unspecific (*n* = 1246), a pattern where no specific chronic conditions were over-represented (see Table 1 for the main characterizing diseases in each pattern, and Additional file 1: Table S1 for the complete list).

**Table 1:** Characterizing diseases with prevalence over 20% within each multimorbidity pattern

Multimorbidity pattern	Characterizing chronic diseases	Prevalence within the pattern (%)
**Unspecific**	-	-
**Psychiatric, respiratory & MSK**	Depression mood disease	36
	Colitis related disease	26
	Asthma	24
	Thyroid disease	24
**Anemia & sensory impairment**	Anemia	31
	Deafness hearing loss	27
	Cataract lens disease	21
	Glaucoma	20
**Cardiometabolic & inflammatory**	Heart failure	75
	Ischemic heart disease	55
	Atrial fibrillation	45
	Anemia	32
	Diabetes	23
	Cerebrovascular disease	21

Chronic diseases with observed/expected ratio ≥ 2 and exclusivity > 25%. MSK: musculoskeletal; COPD: chronic obstructive pulmonary disease. Further details in Table S1

The baseline descriptive characteristics of the study population according to disease patterns are presented in Table 2. Among individuals with multimorbidity, those in the “Unspecific” pattern were more likely to be men, younger, and generally healthier than those in the other multimorbidity patterns. Further, they presented high prevalence of cardiometabolic risk factors, e.g., hypertension (86.3%), and diabetes (9.5%). Individuals in the “Cardiometabolic & inflammatory” pattern were more likely to be older, with lower education level, and with higher number of chronic diseases and drugs compared to those in the other multimorbidity patterns. Regarding physical performance, the prevalence of impaired walking speed and chair stands tests followed a gradient: lowest in the no multimorbidity group, increasing across the “Unspecific”, “Psychiatric, respiratory & musculoskeletal”, “Anemia & sensory impairment” patterns, and peaking in the “Cardiometabolic & Inflammatory” pattern.

**Table 2:** Baseline descriptive characteristics of the study population by disease patterns (*n* = 3112)

	**Total**	**No MM**	**Unspecific**	**Psychiatric, respiratory & MSK**	**Anemia & sensory impairment**	**Cardiometabolic & inflammatory**
	***N* = 3,112**	***N* = 421 (13.5%)**	***N* = 1,246 (40%)**	***N* = 482 (15.4%)**	***N* = 609 (19.6%)**	***N* = 354 (11.4%)**
Sex (female), *n* (%)	1,971 (63.3%)	224 (53.2%)	739 (59.3%)	370 (76.8%)	413 (67.8%)	225 (63.6%)
Age (years), median (IQR)	72 (66;81)	61 (60;67)	67 (61;78)	72 (64;78)	81 (78;90)	84 (78;90)
Education (university), *n* (%)	1,063 (34.3%)	218 (51.9%)	457 (36.7%)	162 (33.9%)	164 (27.1%)	62 (17.7%)
Living in institution, *n* (%)	56 (1.8%)	1 (0.2%)	5 (0.4%)	10 (2.1%)	20 (3.3%)	20 (5.6%)
Alcohol consumption						
no or occasional, *n* (%)	1,069 (34.7%)	67 (16.0%)	333 (26.8%)	190 (40.0%)	282 (47.4%)	197 (56.6%)
light-to-moderate, *n* (%)	1,511 (49.1%)	280 (66.7%)	681 (54.9%)	190 (40.0%)	236 (39.7%)	124 (35.6%)
heavy drinking, *n* (%)	499 (16.2%)	73 (17.4%)	227 (18.3%)	95 (20.0%)	77 (12.9%)	27 (7.8%)
Smoking habit, *n* (%)	452 (14.7%)	74 (17.8%)	188 (15.2%)	95 (20.1%)	57 (9.5%)	38 (10.9%)
Malnutrition (BMI < 18.5 kg/m^2^), *n* (%)	81 (2.6%)	2 (0.5%)	14 (1.1%)	19 (3.9%)	30 (4.9%)	16 (4.5%)
Obesity, *n* (%)	390 (12.5%)	11 (2.6%)	248 (19.9%)	44 (9.1%)	39 (6.4%)	48 (13.6%)
No. of medications, median (IQR)	3 (1;6)	1 (0;2)	2 (1;4)	5 (3;7)	4 (2;6)	7 (5;10)
No. of chronic diseases, median (IQR)	3 (2;5)	1 (1;1)	3 (2;4)	4 (3;5)	5 (4;6)	7 (6;9)
Hypertension, *n* (%)	2,170 (69.7%)	129 (30.6%)	1,075 (86.3%)	261 (54.1%)	467 (76.7%)	238 (67.2%)
Diabetes, *n* (%)	275 (8.8%)	4 (1.0%)	118 (9.5%)	16 (3.3%)	54 (8.9%)	83 (23.4%)
*PHYSICAL PERFORMANCE ASSESSMENT*						
Walking speed test (m/s)						
median (IQR)	1.2 (0.8;1.2)	1.2 (1.2;1.5)	1.2 (1;1.5)	1.0 (0.7;1.2)	0.8 (0.5;1.2)	0.6 (0.3;0.8)
impaired test, *n* (%)	789 (25.9%)	18 (4.3%)	154 (12.5%)	123 (26.0%)	272 (45.9%)	222 (66.1%)
Chair stands test (s)						
median (IQR)	14 (10;24)	11 (9;14)	12 (10;17)	15 (11;37)	19 (13;52)	52 (15;52)
impaired test, *n* (%)	1,288 (42.0%)	58 (13.9%)	386 (31.1%)	224 (47.4%)	375 (62.7%)	245 (72.1%)

*IQR*: Interquartile range, *MM* Multimorbidity, *MSK* Musculoskeletal, *BMI* Body mass index

Missing data: age (*n* = 8) education (*n* = 14), alcohol consumption (*n* = 33), smoking (*n* = 35), walking speed test (*n* = 63), chair stand test (*n* = 42)

Differences across multimorbidity patterns were assessed using Chi-square tests for categorical variables and Kruskal–Wallis rank tests for continuous variables, and results were all statistically significant

**Fig. 1:**
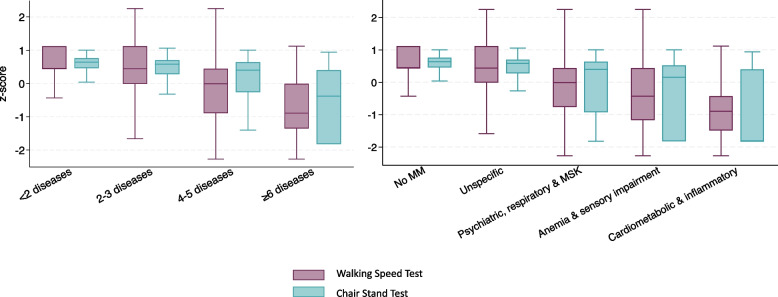
Distribution of the walking speed test (z-score) and chair stand test (z-score) by chronic diseases count and multimorbidity patterns. Box plots show the median (central line) and interquartile range (box) as well as the 2.5th and 97.5th percentiles (whiskers)

Figure 1 shows the distribution of baseline physical performance, operationalized as the standardized average of walking speed and chair stand measures, by number of chronic diseases and multimorbidity patterns. As the number of chronic conditions or the complexity of the multimorbidity pattern increased, overall physical performance tended to worsen. However, a marked variability was observed within all groups, with wide and overlapping interquartile ranges.

### Multimorbidity and longitudinal trajectories of physical performance

Overall, the presence of multimorbidity was associated with accelerated decline in physical performance compared with individuals without multimorbidity. Table 3 reports the β coefficients reflecting the annual change in the combined physical function measures over the 15-year follow-up (corresponding values in the original units of m/s and seconds are presented in Additional file 1: Figure S2 and Table S2; Table S9 reports information on follow-up time) in relation to the number of diseases and disease patterns.

**Table 3:** Associations between chronic diseases count and multimorbidity patterns and annual change (β coefficients) in walking speed test (z-score), chair stand test (z-score) and combined physical function measure (z-score) over the 15-year follow-up (*n* = 3036)

Chronic diseases count				MM Patterns			
2–3 (n = 1117)		4–5 (n = 830)	≥ 6 (n = 672)	Unspecific (n = 1231)	Psychiatric, respiratory & MSK (n = 470)	Anemia & sensory impairment (n = 587)	Cardiometabolic & inflammatory (n = 333)
Combined measure (z-score)							
−0.021 [−0.034,−0.007]		−0.028 [−0.041,−0.014]	−0.059 [−0.086,−0.033]	−0.025 [−0.037,−0.012]	−0.024 [−0.042,−0.006]	−0.043 [−0.063,−0.023]	−0.066 [−0.111,−0.021]
Walking speed test (z-score)							
−0.019 [−0.031,−0.007]		−0.023 [−0.036,−0.009]	−0.051 [−0.079,−0.024]	−0.021 [−0.033,−0.009]	−0.021 [−0.038,−0.004]	−0.040 [−0.061,−0.020]	−0.051 [−0.100,−0.003]
Chair stand test (z-score)							
−0.021 [−0.037,−0.004]		−0.031 [−0.049,−0.013]	−0.070 [−0.100,−0.041]	−0.027 [−0.043,−0.012]	−0.030 [−0.054,−0.007]	−0.043 [−0.071,−0.015]	−0.075 [−0.123,−0.027]

*MM* Multimorbidity, *CI* Confidence interval, *MSK* Musculoskeletal

Models adjusted for age, sex, education, Mini Mental State Examination, Body mass index, and number of medications

Reference group: individuals without multimorbidity (n = 415)

95% confidence intervals in brackets

All results were statistically significant

**Fig. 2:**
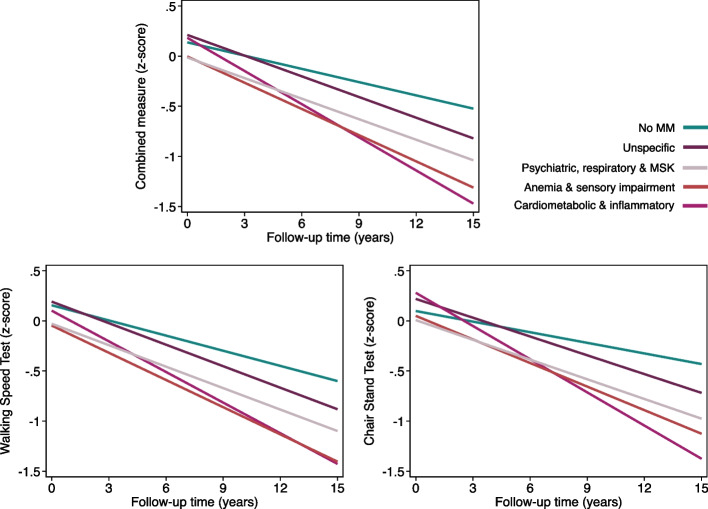
Trajectories of the combined physical function measure (z-score), walking speed test (z-score) and chair stand test (z-score) over 15 years by multimorbidity patterns. Trajectories derived from mixed-effect linear regression models adjusted for baseline age, sex, education, Mini Mental State Examination, Body mass index, and number of medications. Reference group: individuals without multimorbidity. MM: multimorbidity; MSK: musculoskeletal

Figure 2 shows predicted trajectories based on multimorbidity patterns.

A greater number of chronic conditions was associated with a faster decline in physical performance, both for walking speed and chair stand, following a clear gradient in effect size (2–3 conditions: β = –0.021; 4–5 conditions: β = –0.028; ≥ 6 conditions: β = –0.059; all coefficients were statistically significant). In terms of patterns, all groups presented poorer trajectories compared to individuals without multimorbidity. The Cardiometabolic & Inflammatory pattern showed the fastest annual decline across all physical performance measures, with the steepest reduction observed for the chair stand test (β: –0.075, 95% CI: –0.123, –0.027). In contrast, individuals in the Psychiatric, Respiratory & Musculoskeletal pattern had the lowest baseline levels of physical performance, but their decline over time was less pronounced, particularly for walking speed (β: –0.021, 95% CI: –0.038, –0.004). Conversely, those in the Unspecific pattern had a baseline performance comparable to individuals without multimorbidity, yet their trajectories worsened significantly over time, especially for chair stand performance (β: –0.027, 95% CI: –0.043, –0.012).

Differences between the two performance measures were also observed across patterns. In the Anemia & Sensory Impairment pattern, the rate of decline was similar for walking speed (β: –0.040, 95% CI: –0.061, –0.020) and chair stand (β: –0.043, 95% CI: –0.071, –0.015). However, in the Cardiometabolic & Inflammatory pattern, the decline was more pronounced for chair stand (β: –0.075) than for walking speed (β: –0.051).

When restricting the analysis to individuals with multimorbidity and using the Unspecific pattern as the reference group (Additional file 1: Figure S3 and Table S3), most associations were attenuated, with the Cardiometabolic & Inflammatory pattern being associated with an annual decline on the combined measure of − 0.041 (95% CI: − 0.086, 0.003).

Interaction terms for age and sex were not significant. When stratifying by age or sex (Additional file 1: Table S4 and Table S5), no relevant differences were observed in the associations between multimorbidity patterns and physical performance.

### Sensitivity analyses

Results remained consistent after adjusting for number of chronic diseases or institutionalization (Additional file 1: Table S7 and S11). Further, excluding individuals with less than two measures of physical performance tests during follow-up and individuals developing dementia within follow-up yielded results consistent with the main findings (Additional file 1: Figure S4, Table S6, and Table S8). Lastly, results remained consistent after assigning alternative imputed values to participants unable to perform the tests due to physical limitations, with attenuated effect sizes but a persistent gradient across multimorbidity patterns (Additional file 1: Table S14).

## Discussion

This study shows that distinct multimorbidity patterns were differentially associated with long-term trajectories of physical performance in older adults. While a higher number of chronic diseases was linked to a progressively steeper decline in a combined measure of physical function, individuals in the Cardiometabolic & Inflammatory pattern experienced the most pronounced deterioration, particularly in lower limb strength. Notably, these associations remained significant even after adjusting for the number of chronic conditions, suggesting that the combination of specific diseases is linked to physical performance trajectories even after accounting for the overall disease burden. Differences in physical performance trajectories across walking speed and chair stand measures further highlight the heterogeneous relationships between multimorbidity and functional decline. These findings highlight the relevance of multimorbidity patterns in relation to trajectories of physical function throughout aging.

Research efforts to identify replicable and clinically relevant multimorbidity patterns have been increasing in recent years [1, 25, 31, 32]. Despite this growing body of research, considerable heterogeneity remains in the patterns identified, largely due to differences in study populations, healthcare contexts, the range of chronic conditions assessed, and the clustering methodologies applied [33–35]. Nonetheless, systematic reviews have highlighted some consistency, particularly in relation to cardiovascular, musculoskeletal, and neuropsychiatric clusters [32, 36]. In addition, our analysis also identified an Unspecific pattern characterized by the absence of over-expressed diseases in individuals with chronic diseases, which is consistent with previous findings [37, 38]. Specifically, this group exhibited a relatively lower number of chronic conditions but a higher prevalence of cardiometabolic risk factors, suggesting early disease burden [37]. Importantly, evidence indicates that individuals in this group may be at increased risk of progressing toward more complex multimorbidity profiles over time, making them a potentially important target for secondary prevention strategies [37].

While the association of disease combinations and trajectories of physical performance has been previously explored [39–42], only one previous study from the China Health and Retirement Longitudinal Study [26] has investigated data-driven multimorbidity patterns and physical performance to our knowledge. In line with our findings, Yao et al. reported that a higher number of chronic diseases is associated with greater worsening in physical performance. Conversely, in contrast to our observations, individuals in the mental–sensory pattern experienced a more pronounced worsening in physical performance. Nonetheless, direct comparisons between the two studies are hampered due to several methodological differences. First, unlike Yao et al., we excluded individuals with a clinical diagnosis of dementia from our analytical sample to ensure that the results were not affected by severe cognitive impairment. Second, in the study by Yao et al., physical performance was assessed using the handgrip strength test in addition to walking speed. In contrast, our study focused on the chair stands test, a complex measure of physical performance that requires the interplay of muscle strength, balance, and movement coordination. Notably, the chair stands test is a core component of the widely used Short Physical Performance Battery (SPPB) for assessing physical performance [43]. Third, in SNAC-K the ascertainment of chronic conditions is physician-assessed and further integrates diagnostic data from multiple healthcare sources, resulting in an extensive disease list used to derive multimorbidity patterns [25]. Lastly, the 15-year follow-up in the present analysis substantially exceeds the four-year follow-up in Yao et al., further reduces comparability between the studies.

Individuals characterized by the Cardiometabolic & Inflammatory pattern exhibited a faster annual decline in physical performance as measured by objective tests, followed by individuals in the Anemia & Sensory Impairment, Psychiatric, Respiratory & Musculoskeletal, and Unspecific patterns. Further, among individuals with multimorbidity, the Cardiometabolic & Inflammatory pattern showed worse trajectories compared to the Unspecific group, although attenuated. These findings highlight worse physical performance trajectories among individuals with more biologically complex multimorbidity patterns compared to those with simpler patterns. While a higher number of chronic conditions may partly explain these results, the specific composition of disease patterns likely plays an additional role. Indeed, although more complex patterns tended to involve a greater disease count, our analyses showed that these patterns remained independently associated with faster functional decline even after adjusting for total disease burden. This suggests that the presence of specific disease combinations, beyond the count of diseases per se, may have a synergistic detrimental effect on physical performance. Potential mechanisms include overlapping pathophysiological processes such as systemic inflammation, metabolic and endocrine dysfunction, nutritional depletion, and the demands of more complex care regimens, which may contribute to functional loss through treatment burden or care fragmentation [1, 44, 45].

In our sample, the decline in physical performance varied across multimorbidity patterns and presented nuances across the two employed tests. Specifically, the chair stands test exhibited the steepest annual decline, particularly in individuals belonging to the Cardiometabolic & Inflammatory pattern, while the Anemia & Sensory Impairment pattern showed similar longitudinal trajectories for both physical performance tests. These differences may be related to the specific characteristics of each test, which could reflect physical function from different perspectives. The chair stands test, for instance, has been previously considered a measure of physical performance particularly related to cardiovascular health [46–49], which could explain the more pronounced decline seen in individuals within the Cardiometabolic & Inflammatory pattern. In addition, as systemic inflammation has been associated with reduced muscle mass and strength [50, 51], these results may reflect that chronic low-grade inflammation, a common feature in complex multimorbidity [52, 53], can further accelerate sarcopenia-related loss of strength, which is well captured by the chair stands test. Conversely, the walking speed test, beyond lower-limb strength, has been related to cognitive health, with impaired walking speed considered an early sign of cognitive impairment [54]. Notably, our results remained consistent after excluding individuals who developed dementia during follow-up, indicating that these associations reflect physical performance independently of cognitive deterioration.

The findings of this study may have important implications for prevention and clinical practice. First, while the number of chronic conditions remains a relevant indicator of disease burden, a pattern-based approach may offer greater clinical utility for risk stratification. Specific multimorbidity patterns, particularly those characterized by cardiometabolic and inflammatory diseases, were more strongly associated with poorer trajectories, suggesting that the composition of diseases provides critical information beyond simple disease count. Similar associations have been observed for other outcomes such as dementia, depression, frailty, disability, institutionalization, and unplanned hospitalizations in SNAC-K [18, 20, 22–24, 55]. Although methodological consensus on how to define multimorbidity patterns is still evolving, recognizing and employing clinically meaningful disease combinations in clinical decision-making may offer a better framework to address the challenges of multimorbidity—especially given the limitations of the traditional definition (i.e., 2 + diseases), which has failed to guide effective interventions [56, 57]. Second, these findings support the need for systematic assessment of physical performance in all individuals with multimorbidity, including those with lower clinical complexity. Objective measures such as walking speed and chair stand tests are easy-to-perform and valuable for detecting early decline and tailoring care. Given the strong evidence supporting the effectiveness of interventions, particularly physical exercise, in mitigating aging-related physical decline [58–63], regular monitoring of physical function in older people with varying degrees of multimorbidity should be emphasized. This would enable more personalized and timely interventions, helping to preserve functional ability and delay further physical decline.

The strengths of this study include: a large study population, a 15-year follow-up period, the use of objective measures of physical performance, the comprehensive clinical evaluation that allows for the chronic disease assessment, the rigorous clinically driven methodology that was used to operationalize multimorbidity, and the definition of data-driven multimorbidity patterns.

However, several limitations of this study should be acknowledged and discussed. First, dropout and death during follow-up differed across multimorbidity patterns and were likely to be associated with faster decline in physical function, potentially underestimating the association between disease patterns and physical performance. To mitigate this issue, IPWs were applied in all analyses. Second, the relatively long intervals between follow-up waves may have limited our ability to detect more subtle changes in physical performance; still, the use of two sensitive physical function tests likely mitigated this issue. Third, we did not account for disease severity, although we controlled for BMI and number of drugs. Nevertheless, sensitivity analyses adjusting for total disease count (a gross indicator of clinical complexity that tends to correlate with the severity of individual diseases) confirmed the robustness of our findings. Fourth, the inclusion of participants with less than 2 assessments may have biased the association between patterns and physical decline due to selective dropout of the frailest individuals. However, limiting the analysis to individuals with at least one additional assessment of physical performance during follow-up confirmed the associations. Fifth, the exclusion of individuals with dementia at baseline limits the generalizability of our findings to cognitively intact older adults. Nevertheless, this approach was adopted to ensure that the assessment of physical performance was not compromised by severe cognitive impairment. Sixth, assigning imputed values to participants unable to perform the tests due to physical limitations may have influenced effect estimates, potentially attenuating or amplifying differences across multimorbidity patterns. Nevertheless, imputing non-completion of physical performance tests using the 90th and 75th percentile values of the study population, which are consistent with normative values previously reported for older populations [64], did not alter the pattern of results. Last, participants in the SNAC-K study are relatively fit and healthy individuals, which may limit the generalizability of our findings to other populations of older adults.

## Conclusions

Our findings suggest that multimorbidity patterns are differentially associated with a faster decline in physical performance, highlighting the value of considering disease combinations (beyond the overall disease count) when assessing functional trajectories in older adults. In particular, patterns involving cardiometabolic and inflammatory conditions are linked to more rapid deterioration. These results underscore the importance of personalized monitoring and support targeted interventions tailored to specific multimorbidity profiles to help preserve physical function in aging populations.

## Supplementary Information

Additional file 1. Supplementary Figures and Tables (Figures S1–S4; Tables S1–S15). Fig. S1. Flow chart of study population and participation over 15 years. Fig. S2. Walking speed and chair stand trajectories over 15 years by multimorbidity patterns. Fig. S3. Trajectories of combined physical function, walking speed, and chair stand z-scores over 15 years by multimorbidity patterns (Unspecific reference). Fig. S4. Physical function, walking speed, and chair stand z-score trajectories by multimorbidity patterns after excluding individuals with <2 performance measures. Table S1. Multimorbidity pattern characterization. Table S2. Chronic disease count, multimorbidity patterns, and annual change in walking speed and chair stand tests over 15 years. Table S3. Multimorbidity patterns and annual change in physical function z-scores over 15 years (Unspecific reference). Table S4. Multimorbidity patterns and annual change in physical function z-scores by sex. Table S5. Multimorbidity patterns and annual change in physical function z-scores by age group. Table S6. Chronic disease count, multimorbidity patterns, and annual change in physical function z-scores after excluding individuals with <2 performance measures. Table S7. Multimorbidity patterns and annual change in physical function z-scores additionally adjusted for number of chronic diseases. Table S8. Chronic disease count, multimorbidity patterns, and annual change in physical function z-scores after excluding dementia cases within 6 years. Table S9. Follow-up of the study population by multimorbidity patterns. Table S10. Multimorbidity patterns and annual change in physical function z-scores with and without inverse probability weighting. Table S11. Multimorbidity patterns and annual change in combined physical function with additional adjustment for institutionalization. Table S12. Walking speed values imputed due to inability to perform the test by multimorbidity patterns and wave. Table S13. Chair stand values imputed due to inability to perform the test by multimorbidity patterns and wave. Table S14. Multimorbidity patterns and annual change in walking speed and chair stand time with inability imputed using 90th and 75th percentiles. Table S15. STROBE checklist for cohort studies

## Data Availability

SNAC-K data (http://www.snac-k.se/) can be accessed by the scientific community upon approval from the SNAC-K management and maintenance committee, and applications can be submitted to Maria Wahlberg (Maria.Wahlberg@ki.se) at the Aging Research Center, Karolinska Institutet.

## Abbreviations

ADL: Activities of daily living
BMI: Body mass index
BIC: Bayesian information criterion
CI: Confidence interval
ICD-10: International Classification of Diseases, 10th Revision
IADL: Instrumental activities of daily living
IQR: Interquartile range
IPW: Inverse probability weighting
LCA: Latent class analysis
MLTC: Multiple long-term conditions
MMSE: Mini Mental State Examination
MSK: Musculoskeletal
O/E: Observed-to-expected ratio
SNAC-K: Swedish National Study on Aging and Care in Kungsholmen
SPPB: Short Physical Performance Battery
WHO: World Health Organization
