# Supplementary material for: Multimorbidity patterns and 15-year trajectories of physical performance: a population-based study
Source: BMC Med. 2026 Mar 25;24:216. doi: 10.1186/s12916-026-04828-6 (PMC13063542; doi:10.1186/s12916-026-04828-6)
Supplement: Supplementary file 1 — Additional file 1. Supplementary Figures and Tables (Figures S1–S4; Tables S1–S15). Fig. S1. Flow chart of study population and participation over 15 years. Fig. S2. Walking speed and chair stand trajectories over 15 years by multimorbidity patterns. Fig. S3. Trajectories of combined physical function, walking speed, and chair stand z-scores over 15 years by multimorbidity patterns (Unspecific reference). Fig. S4. Physical function, walking speed, and chair stand z-score trajectories by multimorbidity patterns after excluding individuals with <2 performance measures. Table S1. Multimorbidity pattern characterization. Table S2. Chronic disease count, multimorbidity patterns, and annual change in walking speed and chair stand tests over 15 years. Table S3. Multimorbidity patterns and annual change in physical function z-scores over 15 years (Unspecific reference). Table S4. Multimorbidity patterns and annual change in physical function z-scores by sex. Table S5. Multimorbidity patterns and annual change in physical function z-scores by age group. Table S6. Chronic disease count, multimorbidity patterns, and annual change in physical function z-scores after excluding individuals with <2 performance measures. Table S7. Multimorbidity patterns and annual change in physical function z-scores additionally adjusted for number of chronic diseases. Table S8. Chronic disease count, multimorbidity patterns, and annual change in physical function z-scores after excluding dementia cases within 6 years. Table S9. Follow-up of the study population by multimorbidity patterns. Table S10. Multimorbidity patterns and annual change in physical function z-scores with and without inverse probability weighting. Table S11. Multimorbidity patterns and annual change in combined physical function with additional adjustment for institutionalization. Table S12. Walking speed values imputed due to inability to perform the test by multimorbidity patterns and wave. Table S13. Chair stand values imp [file 12916_2026_4828_MOESM1_ESM.docx]

TABLE OF CONTENTS

[Figure S1. Flow chart of study population definition and participation over 15 years. 4](#_Toc221021349)

[Table S1. Multimorbidity pattern characterization. 5](#_Toc221021350)

[Figure S2. Trajectories of the walking speed test (m/s) and chair stand test (s) over 15 years by multimorbidity patterns. 12](#_Toc221021351)

[Table S2. Associations between chronic diseases count and multimorbidity patterns and annual change in walking speed test (m/s) and chair stand test (s) over the 15-year follow-up (n=3036). 13](#_Toc221021352)

[Figure S3. Trajectories of the combined physical function measure (z-score), walking speed test (z-score) and chair stand test (z-score) over 15 years by multimorbidity patterns with the Unspecific pattern as reference group (n=2621). 14](#_Toc221021353)

[Table S3. Associations between multimorbidity patterns and annual change (β coefficients) in walking speed test (z-score), chair stand test (z-score) and combined physical function measure (z-score) over the 15-year follow-up with the Unspecific pattern as reference group (n=2621). 15](#_Toc221021354)

[Table S4. Associations between multimorbidity patterns and annual change (β coefficients) in walking speed test (z-score), chair stand test (z-score) and combined physical function measure (z-score) over the 15-year follow-up in female (n=1917) and male (n=1121). 16](#_Toc221021355)

[Table S5. Associations between multimorbidity patterns and annual change (β coefficients) in walking speed test (z-score), chair stand test (z-score) and combined physical function measure (z-score) over the 15-year follow-up in individuals aged <78 years (n=1735) and ≥ 78 years (n=1303). 17](#_Toc221021356)

[Figure S4. Trajectories of the combined physical function measure (z-score), walking speed test (z-score) and chair stand test (z-score) overs by multimorbidity patterns (n=2258) after excluding individuals with less than two measures of physical performance tests during follow-up (n=854). 18](#_Toc221021357)

[Table S6. Associations between chronic diseases count and multimorbidity patterns and annual change (β coefficients) in walking speed test (z-score), chair stand test (z-score) and combined physical function measure (z-score) over the 15-year follow-up after excluding individuals with less than two measures of physical performance tests during follow-up (n=854). 19](#_Toc221021358)

[Table S7. Associations between multimorbidity patterns and annual change (β coefficients) in walking speed test (z-score), chair stand test (z-score) and combined physical function measure (z-score) over the 15-year follow-up adjusted for the number of chronic disease in addition to age, sex, education, Mini Mental State Examination, Body mass index, and number of medications 20](#_Toc221021359)

[Table S8. Associations between chronic diseases count and multimorbidity patterns and annual change (β coefficients) in walking speed test (z-score), chair stand test (z-score) and combined physical function measure (z-score) over the 15-year follow-up after excluding individuals who developed dementia within the first 6 years of follow-up. 21](#_Toc221021360)

[Table S9. Follow-up of the study population by disease patterns (n=3112). 22](#_Toc221021361)

[Table S10. Associations between multimorbidity patterns and annual change (β coefficients) in walking speed test (z-score), chair stand test (z-score) and combined physical function measure (z-score) over the 15-year follow-up (n=3036) with and without IPW. 23](#_Toc221021362)

[Table S11. Associations between multimorbidity patterns and annual change (β coefficients) in the combined physical function measure (z-score) over the 15-year follow-up (n=3036) with extra-adjustment for institutionalization. 24](#_Toc221021363)

[Table S12. Numbers of walking speed test values imputed due to inability to perform the test across multimorbidity patterns, by wave. 25](#_Toc221021364)

[Table S13. Distribution of chair stands test values imputed due to inability to perform the test across multimorbidity patterns, by wave. 26](#_Toc221021365)

[Table S14 . Associations between multimorbidity patterns and 15-year annual change in walking speed (m/s) and chair stand time (s), with inability to perform imputed using the 90^th^ and 75^th^ percentile values of the study population. 27](#_Toc221021366)

[Table S15. STROBE statement checklist for cohort studies. 28](#_Toc221021367)

Figure S1. Flow chart of study population definition and participation over 15 years.


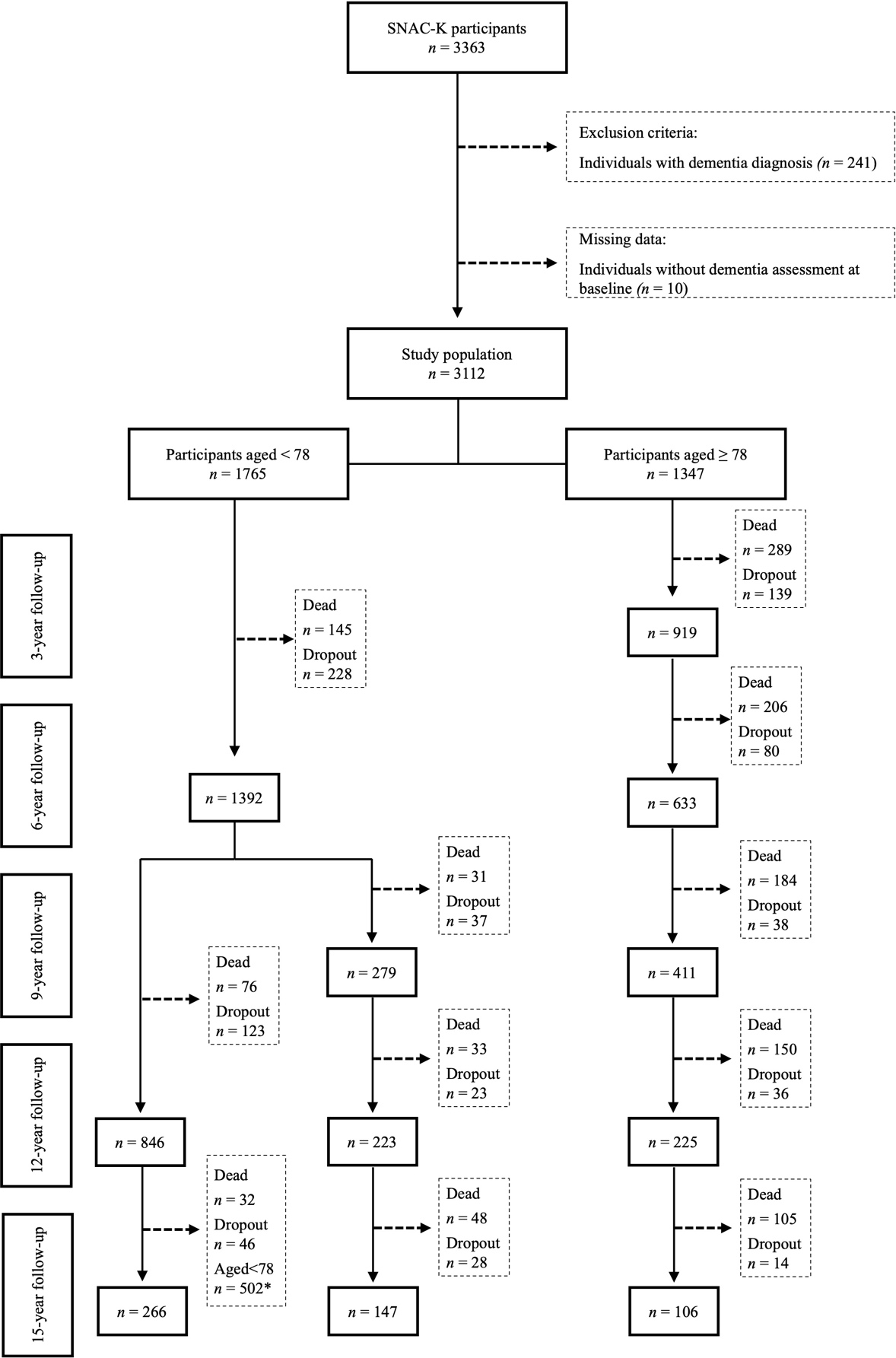


* Participants who were younger than 78 years at the 15-year follow-up were not eligible for assessment. In accordance with the study design, they were instead followed up during the seventh wave (18-year follow-up).

Table S1. Multimorbidity pattern characterization.

| **Multimorbidity pattern** | **Chronic diseases** | **Prevalence within the pattern (%)** | **Exclusivity (%)** | **O/E** |
| --- | --- | --- | --- | --- |
| Unspecific | Anemia | 1 | 5 | 0.10 |
|  | Asthma | 4 | 23 | 0.49 |
|  | Atrial fibrillation | 4 | 18 | 0.39 |
|  | Autoimmune diseases | 3 | 28 | 0.61 |
|  | Blindness, visual loss | <1 | <1 | <0.01 |
|  | Bradycardias and conduction diseases | <1 | 8 | 0.18 |
|  | COPD, emphysema and chronic bronchitis | 2 | 14 | 0.31 |
|  | Cardiac valve diseases | 1 | 15 | 0.33 |
|  | Cataract and other lens diseases | 1 | 6 | 0.13 |
|  | Cerebrovascular diseases | 4 | 22 | 0.48 |
|  | Chronic kidney diseases | 23 | 28 | 0.61 |
|  | Colitis and related diseases | 2 | 7 | 0.16 |
|  | Deafness and hearing loss | 3 | 13 | 0.28 |
|  | Depression and mood diseases | 2 | 9 | 0.19 |
|  | Diabetes | 9 | 44 | 0.94 |
|  | Dorsopathies | 3 | 20 | 0.44 |
|  | Dyslipidemia | 68 | 59 | 1.27 |
|  | Esophagus, stomach and duodenum diseases | 3 | 30 | 0.65 |
|  | Glaucoma | 1 | 7 | 0.16 |
|  | Heart failure | 1 | 4 | 0.09 |
|  | Hypertension | 86 | 53 | 1.14 |
|  | Inflammatory arthropathies | 2 | 16 | 0.34 |
|  | Ischemic heart diseases | 11 | 32 | 0.68 |
|  | Migraine and facial pain syndromes | 2 | 37 | 0.79 |
|  | Neurotic, stress-related and somatoform diseases | <1 | <1 | <0.01 |
|  | Obesity | 20 | 65 | 1.41 |
|  | Osteoarthritis and other degenerative joint diseases | 12 | 38 | 0.83 |
|  | Osteoporosis | <1 | 0 | <0.01 |
|  | Other MSK joint diseases | 5 | 35 | 0.77 |
|  | Other cardiovascular diseases | 1 | 11 | 0.23 |
|  | Other eye diseases | <1 | 1 | 0.01 |
|  | Other genitourinary diseases | 3 | 45 | 0.97 |
|  | Other neurological diseases | 1 | 20 | 0.44 |
|  | Prostate diseases | 4 | 44 | 0.95 |
|  | Sleep diseases | 2 | 43 | 0.93 |
|  | Solid neoplasms | 8 | 37 | 0.81 |
|  | Thyroid diseases | 7 | 30 | 0.65 |
| **Psychiatric, respiratory & MSK** | Anemia | 6 | 8 | 0.47 |
|  | **Asthma** | **24** | **59** | **3.31** |
|  | Atrial fibrillation | 3 | 6 | 0.32 |
|  | Autoimmune diseases | 5 | 19 | 1.04 |
|  | Blindness, visual loss | <1 | 1 | 0.05 |
|  | Bradycardias and conduction diseases | 1 | 10 | 0.57 |
|  | **COPD, emphysema and chronic bronchitis** | **13** | **40** | **2.24** |
|  | Cardiac valve diseases | 1 | 4 | 0.21 |
|  | Cataract and other lens diseases | 2 | 5 | 0.30 |
|  | Cerebrovascular diseases | 4 | 8 | 0.45 |
|  | Chronic kidney diseases | 27 | 12 | 0.69 |
|  | **Colitis and related diseases** | **26** | **38** | **2.14** |
|  | Deafness and hearing loss | 5 | 8 | 0.47 |
|  | **Depression and mood diseases** | **36** | **65** | **3.64** |
|  | Diabetes | 3 | 6 | 0.33 |
|  | **Dorsopathies** | **18** | **43** | **2.41** |
|  | Dyslipidemia | 47 | 16 | 0.88 |
|  | **Esophagus, stomach and duodenum diseases** | **11** | **37** | **2.05** |
|  | Glaucoma | 2 | 5 | 0.27 |
|  | Heart failure | <1 | <1 | 0.02 |
|  | Hypertension | 54 | 13 | 0.71 |
|  | Inflammatory arthropathies | 4 | 15 | 0.84 |
|  | Ischemic heart diseases | 4 | 4 | 0.25 |
|  | Migraine and facial pain syndromes | 4 | 28 | 1.56 |
|  | **Neurotic, stress-related and somatoform diseases** | **16** | **82** | **4.55** |
|  | Obesity | 9 | 12 | 0.65 |
|  | Osteoarthritis and other degenerative joint diseases | 22 | 27 | 1.49 |
|  | **Osteoporosis** | **17** | **40** | **2.24** |
|  | Other MSK joint diseases | 10 | 25 | 1.41 |
|  | Other cardiovascular diseases | 2 | 10 | 0.54 |
|  | Other eye diseases | 3 | 8 | 0.45 |
|  | Other genitourinary diseases | 1 | 9 | 0.50 |
|  | **Other neurological diseases** | **5** | **39** | **2.18** |
|  | Prostate diseases | 1 | 6 | 0.31 |
|  | **Sleep diseases** | **6** | **40** | **2.25** |
|  | Solid neoplasms | 7 | 12 | 0.67 |
|  | **Thyroid diseases** | **24** | **37** | **2.05** |
| **Anemia & sensory impairment** | **Anemia** | **31** | **54** | **2.40** |
|  | Asthma | 1 | 4 | 0.16 |
|  | Atrial fibrillation | 9 | 20 | 0.86 |
|  | Autoimmune diseases | 6 | 26 | 1.14 |
|  | **Blindness, visual loss** | **13** | **68** | **3.01** |
|  | Bradycardias conduction diseases | <1 | 0 | <0.01 |
|  | COPD, emphysema and chronic bronchitis | 2 | 9 | 0.41 |
|  | Cardiac valve diseases | 3 | 21 | 0.91 |
|  | **Cataract and other lens diseases** | **21** | **77** | **3.39** |
|  | Cerebrovascular diseases | 12 | 34 | 1.49 |
|  | Chronic kidney diseases | 58 | 34 | 1.52 |
|  | Colitis and related diseases | 16 | 30 | 1.34 |
|  | **Deafness and hearing loss** | **27** | **55** | **2.41** |
|  | Depression and mood diseases | 5 | 11 | 0.50 |
|  | Diabetes | 9 | 20 | 0.88 |
|  | Dorsopathies | 6 | 17 | 0.75 |
|  | Dyslipidemia | 35 | 15 | 0.67 |
|  | Esophagus, stomach and duodenum diseases | 3 | 14 | 0.64 |
|  | **Glaucoma** | **20** | **72** | **3.17** |
|  | Heart failure | 2 | 3 | 0.15 |
|  | Hypertension | 77 | 23 | 1.01 |
|  | Inflammatory arthropathies | 7 | 33 | 1.47 |
|  | Ischemic heart diseases | 15 | 21 | 0.92 |
|  | Migraine and facial pain syndromes | 1 | 10 | 0.45 |
|  | Neurotic, stress-related and somatoform diseases | 1 | 8 | 0.34 |
|  | Obesity | 6 | 10 | 0.45 |
|  | Osteoarthritis and other degenerative joint diseases | 13 | 19 | 0.85 |
|  | Osteoporosis | 12 | 37 | 1.62 |
|  | Other MSK joint diseases | 6 | 19 | 0.86 |
|  | Other cardiovascular diseases | 2 | 14 | 0.60 |
|  | **Other eye diseases** | **19** | **73** | **3.23** |
|  | Other genitourinary diseases | 4 | 31 | 1.36 |
|  | Other neurological diseases | 3 | 32 | 1.42 |
|  | Prostate diseases | 6 | 29 | 1.29 |
|  | Sleep diseases | <1 | 1 | 0.07 |
|  | Solid neoplasms | 17 | 38 | 1.68 |
|  | Thyroid diseases | 9 | 18 | 0.81 |
| **Cardiometabolic & inflammatory** | **Anemia** | **32** | **33** | **2.48** |
|  | Asthma | 8 | 14 | 1.10 |
|  | **Atrial fibrillation** | **45** | **57** | **4.30** |
|  | **Autoimmune diseases** | **11** | **27** | **2.08** |
|  | **Blindness, visual loss** | **10** | **31** | **2.36** |
|  | **Bradycardias conduction diseases** | **14** | **81** | **6.18** |
|  | **COPD, emphysema and chronic bronchitis** | **16** | **36** | **2.75** |
|  | **Cardiac valve diseases** | **13** | **60** | **4.58** |
|  | Cataract and other lens diseases | 6 | 12 | 0.90 |
|  | **Cerebrovascular diseases** | **21** | **36** | **2.74** |
|  | Chronic kidney diseases | 72 | 25 | 1.88 |
|  | Colitis and related diseases | 22 | 24 | 1.83 |
|  | Deafness and hearing loss | 21 | 24 | 1.84 |
|  | Depression and mood diseases | 11 | 15 | 1.11 |
|  | **Diabetes** | **23** | **31** | **2.33** |
|  | Dorsopathies | 11 | 19 | 1.48 |
|  | Dyslipidemia | 42 | 10 | 0.78 |
|  | Esophagus, stomach and duodenum diseases | 7 | 19 | 1.42 |
|  | Glaucoma | 8 | 16 | 1.24 |
|  | **Heart failure** | **75** | **92** | **7.00** |
|  | Hypertension | 67 | 12 | 0.89 |
|  | **Inflammatory arthropathies** | **13** | **36** | **2.71** |
|  | **Ischemic heart diseases** | **55** | **43** | **3.27** |
|  | Migraine and facial pain syndromes | 5 | 25 | 1.90 |
|  | Neurotic, stress-related and somatoform diseases | 3 | 11 | 0.83 |
|  | Obesity | 14 | 13 | 0.96 |
|  | Osteoarthritis and other degenerative joint diseases | 18 | 16 | 1.18 |
|  | Osteoporosis | 13 | 23 | 1.75 |
|  | Other MSK joint diseases | 10 | 20 | 1.51 |
|  | **Other cardiovascular diseases** | **19** | **66** | **5.02** |
|  | Other eye diseases | 8 | 18 | 1.38 |
|  | Other genitourinary diseases | 3 | 15 | 1.17 |
|  | Other neurological diseases | 1 | 8 | 0.64 |
|  | Prostate diseases | 8 | 21 | 1.62 |
|  | Sleep diseases | 3 | 15 | 1.13 |
|  | Solid neoplasms | 10 | 13 | 0.96 |
|  | Thyroid diseases | 13 | 15 | 1.13 |

O/E = Observed over expected ratio; MSK: Musculoskeletal; COPD: Chronic Obstructive Pulmonary Disease
Bold indicates over-represented chronic diseases within each pattern, defined by an exclusivity ≥25% and an O/E ratio ≥2.

Figure S2. Trajectories of the walking speed test (m/s) and chair stand test (s) over 15 years by multimorbidity patterns.

Trajectories derived from mixed-effect linear regression models adjusted for baseline age, sex and education, Mini Mental State Examination, Body mass index, and number of medications. Reference group: individuals without multimorbidity. MM: multimorbidity; MSK: musculoskeletal.

Table S2. Associations between chronic diseases count and multimorbidity patterns and annual change in walking speed test (m/s) and chair stand test (s) over the 15-year follow-up (*n*=3036).

|  |  | **Chronic diseases count** | | |  | **MM Patterns** | | | |
| --- | --- | --- | --- | --- | --- | --- | --- | --- | --- |
|  |  | **2-3**  **(***n***=1117)** | **4-5**  **(***n***=830)** | **≥6**  **(***n***=672)** |  | **Unspecific**  **(***n***=1231)** | **Psychiatric, respiratory & MSK**  **(***n***=470)** | **Anemia & sensory impairment**  **(***n***=587)** | **Cardiometabolic & inflammatory**  **(***n***=333)** |
| **Walking speed test (m/s)** | |  |  |  |  |  |  |  |  |
|  |  | -0.008  [-0.014,-0.003] | -0.010  [-0.016,-0.004] | -0.023  [-0.035,0.011] |  | -0.009  [-0.015,-0.004] | -0.009  [-0.017,-0.002] | -0.018  [-0.027,-0.009] | -0.023  [-0.044,-0.001] |
| **Chair stand test**  **(s)** | |  |  |  |  |  |  |  |  |
|  |  | 0.344  [0.066,0.622] | 0.523  [0.216,0.824] | 1.171  [0.683,1.660] |  | 0.454  [0.195,0.713] | 0.505  [0.109,0.900] | 0.719  [0.250,0.188] | 1.252  [0.448,2.005] |

MM: multimorbidity; CI: confidence interval; MSK: musculoskeletal.

Models adjusted for age, sex, education, Mini Mental State Examination, Body mass index, and number of medications.

Reference group: individuals without multimorbidity (*n***=**415)

95% confidence intervals in brackets;

All results were statistically significant.

This table shows the results from fitting linear mixed effect models with longitudinal physical function test as outcomes and baseline chronic diseases count or baseline multimorbidity patterns as exposures. β-coefficients are interpreted as 1) walking speed test decline/year per each chronic disease count group or multimorbidity pattern compared to the no multimorbidity group, and 2) chair stand test time increase/year per each per each chronic diseases count group or multimorbidity pattern compared to the no multimorbidity group.

Figure S3. Trajectories of the combined physical function measure (z-score), walking speed test (z-score) and chair stand test (z-score) over 15 years by multimorbidity patterns with the Unspecific pattern as reference group (*n*=2621).

Trajectories derived from mixed-effect linear regression models adjusted for baseline age, sex, education, Mini Mental State Examination, Body mass index, and number of medications. Reference group: individuals in MM pattern “Unspecific” (*n***=**345). MM: multimorbidity; MSK: musculoskeletal.

Table S3. Associations between multimorbidity patterns and annual change (β coefficients) in walking speed test (z-score), chair stand test (z-score) and combined physical function measure (z-score) over the 15-year follow-up with the Unspecific pattern as reference group (*n*=2621).

|  |  | **MM Patterns** | | |
| --- | --- | --- | --- | --- |
|  |  | **Psychiatric, respiratory & MSK**  **(***n***=470)** | **Anemia & sensory impairment**  **(***n***=587)** | **Cardiometabolic & inflammatory**  **(***n***=333)** |
| **Combined measure**  **(z-score)** | |  |  |  |
|  |  | 0.000 [-0.016,0.017] | -0.018 [-0.037,0.001] | -0.041 [-0.086,0.003] |
| **Walking speed test**  **(z-score)** | |  |  |  |
|  |  | 0.000 [-0.015,0.015] | -0.019 [-0.037,-0.001]* | -0.031 [-0.079,0.017] |
| **Chair stand test**  **(z-score)** | |  |  |  |
|  |  | -0.003 [-0.026,0.020] | -0.016 [-0.043,0.012] | -0.047 [-0.096,-0.001]* |

MM: multimorbidity; CI: confidence interval; MSK: musculoskeletal.

Models adjusted for age, sex, education, Mini Mental State Examination, Body mass index, and number of medications.

Reference group: individuals in MM pattern “Unspecific” (*n***=**1231)

95% confidence intervals in brackets;

* results statistically significant

Table S4. Associations between multimorbidity patterns and annual change (β coefficients) in walking speed test (z-score), chair stand test (z-score) and combined physical function measure (z-score) over the 15-year follow-up in female (n=1917) and male (n=1121).

|  |  | **Female** | | | |  | **Male** | | | |
| --- | --- | --- | --- | --- | --- | --- | --- | --- | --- | --- |
|  |  | **Unspecific**  **(***n***=739)** | **Psychiatric, respiratory & MSK**  **(***n***= 370)** | **Anemia & sensory impairment**  **(***n***= 413)** | **Cardiometabolic & inflammatory**  **(***n***= 225)** |  | **Unspecific**  **(***n***= 507)** | **Psychiatric, respiratory & MSK**  **(***n***= 112)** | **Anemia & sensory impairment**  **(***n***= 196)** | **Cardiometabolic & inflammatory**  **(***n***= 129)** |
| **Combined measure**  **(z-score)** | |  |  |  |  |  |  |  |  |  |
|  |  | -0.025*  [-0.044,-0.006] | -0.022*  [-0.042,-0.001] | -0.043*  [-0.068,-0.019] | -0.081*  [-0.124,-0.037] |  | -0.026*  [-0.042,-0.010] | -0.033  [-0.071,0.005] | -0.038*  [-0.063,-0.014] | -0.015  [-0.056,0.025] |

CI: confidence interval; MSK: musculoskeletal.

Models adjusted for age, education, Mini Mental State Examination, Body mass index, and number of medications.

Reference group: individuals without multimorbidity (*n***=**415)

95% confidence intervals in brackets;

* results statistically significant

Table S5. Associations between multimorbidity patterns and annual change (β coefficients) in walking speed test (z-score), chair stand test (z-score) and combined physical function measure (z-score) over the 15-year follow-up in individuals aged <78 years (n=1735) and ≥ 78 years (n=1303).

|  |  | **< 78 years** | | | |  | **≥ 78 years** | | | |
| --- | --- | --- | --- | --- | --- | --- | --- | --- | --- | --- |
|  |  | **Unspecific**  **(***n***=873)** | **Psychiatric, respiratory & MSK**  **(***n***=304)** | **Anemia & sensory impairment**  **(***n***=144)** | **Cardiometabolic & inflammatory**  **(***n***=59)** |  | **Unspecific**  **(***n***= 373)** | **Psychiatric, respiratory & MSK**  **(***n***= 178)** | **Anemia & sensory impairment**  **(***n***= 465)** | **Cardiometabolic & inflammatory**  **(***n***= 295)** |
| **Combined measure**  **(z-score)** | |  |  |  |  |  |  |  |  |  |
|  |  | -0.012*  [-0.023,-0.000] | -0.030*  [-0.049,-0.010] | -0.049*  [-0.080,-0.018] | -0.008  [-0.044,0.028] |  | -0.005  [-0.050,0.039] | 0.044  [-0.007,0.095] | 0.024  [-0.021,0.068] | -0.014  [-0.067,0.040] |

CI: confidence interval; MSK: musculoskeletal.

Models adjusted for sex, education, Mini Mental State Examination, Body mass index, and number of medications.

Reference group: individuals without multimorbidity (*n***=**415)

95% confidence intervals in brackets;

* results statistically significant

Figure S4. Trajectories of the combined physical function measure (z-score), walking speed test (z-score) and chair stand test (z-score) overs by multimorbidity patterns (*n*=2258) after excluding individuals with less than two measures of physical performance tests during follow-up (*n*=854).

Trajectories derived from mixed-effect linear regression models adjusted for baseline age, sex, education, Mini Mental State Examination, Body mass index, and number of medications. Reference group: individuals without multimorbidity. MM: multimorbidity; MSK: musculoskeletal.

Table S6. Associations between chronic diseases count and multimorbidity patterns and annual change (β coefficients) in walking speed test (z-score), chair stand test (z-score) and combined physical function measure (z-score) over the 15-year follow-up after excluding individuals with less than two measures of physical performance tests during follow-up (n=854).

|  |  | **Chronic diseases count** | | | |  | | **MM Patterns** | | | | |
| --- | --- | --- | --- | --- | --- | --- | --- | --- | --- | --- | --- | --- |
|  |  | **2-3**  **(***n***=848)** | **4-5**  **(***n***=623)** | **≥6**  **(***n***=420)** |  | | **Unspecific**  **(***n***=969)** | | **Psychiatric, respiratory & MSK**  **(***n***=349)** | **Anemia & sensory impairment**  **(***n***=398)** | **Cardiometabolic & inflammatory**  **(***n***=176)** |  |
| **Combined measure**  **(z-score)** | |  |  |  |  | |  | |  |  |  |  |
|  |  | -0.024  [-0.038,-0.010] | -0.030  [-0.044,-0.017] | -0.067  [-0.095,-0.039] |  | | -0.026  [-0.039,-0.014] | | -0.028  [-0.046,-0.010] | -0.047  [-0.066,-0.028] | -0.079  [-0.123,-0.035] |  |
| **Walking speed test**  **(z-score)** | |  |  |  |  | |  | |  |  |  |  |
|  |  | -0.023  [-0.035,-0.010] | -0.026  [-0.040,-0.012] | -0.059  [-0.087,-0.030] |  | | -0.023  [-0.036,-0.011] | | -0.025  [-0.043,-0.008] | -0.044  [-0.064,-0.024] | -0.064  [-0.112,-0.015] |  |
| **Chair stand test**  **(z-score)** | |  |  |  |  | |  | |  |  |  |  |
|  |  | -0.024  [-0.041,-0.007] | -0.035  [-0.053,-0.016] | -0.080  [-0.110,-0.049] |  | | -0.030  [-0.046,-0.014] | | -0.035  [-0.059,-0.010] | -0.048  [-0.075,-0.021] | -0.091  [-0.137,-0.044] |  |

MM: multimorbidity; CI: confidence interval; MSK: musculoskeletal.

Models adjusted for age, sex, education, Mini Mental State Examination, Body mass index, and number of medications.

Reference group: individuals without multimorbidity (*n***=**345)

95% confidence intervals in brackets;

All results were statistically significant.

Table S7. Associations between multimorbidity patterns and annual change (β coefficients) in walking speed test (z-score), chair stand test (z-score) and combined physical function measure (z-score) over the 15-year follow-up adjusted for the number of chronic disease in addition to age, sex, education, Mini Mental State Examination, Body mass index, and number of medications

|  |  | **Reference group “No MM”** | | | |  | **Reference group “Unspecific”** | | |
| --- | --- | --- | --- | --- | --- | --- | --- | --- | --- |
|  |  | **Unspecific**  **(***n***=1231)** | **Psychiatric, respiratory & MSK**  **(***n***=470)** | **Anemia & sensory impairment**  **(***n***=587)** | **Cardiometabolic & inflammatory**  **(***n***=333)** |  | **Psychiatric, respiratory & MSK**  **(***n***=470)** | **Anemia & sensory impairment**  **(***n***=587)** | **Cardiometabolic & inflammatory**  **(***n***=333)** |
| **Combined measure**  **(z-score)** | |  |  |  |  |  |  |  |  |
|  |  | -0.024*  [-0.037,-0.012] | -0.024*  [-0.046,-0.006] | -0.041*  [-0.059,-0.023] | -0.066*  [-0.110,-0.021] |  | 0.001  [-0.017,0.017] | -0.016  [-0.033,0.001] | -0.041  [-0.085,0.003] |
| **Walking speed test**  **(z-score)** | |  |  |  |  |  |  |  |  |
|  |  | -0.021*  [-0.033,-0.009] | -0.021*  [-0.038,-0.003] | -0.038*  [-0.057,-0.020] | -0.051*  [-0.099,-0.003] |  | 0.000  [-0.015,0.015] | -0.017*  [-0.034,-0.001] | -0.031  [-0.078,0.013] |
| **Chair stand test**  **(z-score)** | |  |  |  |  |  |  |  |  |
|  |  | -0.027*  [-0.042,-0.011] | -0.030*  [-0.053,-0.006] | -0.040*  [-0.066,-0.015] | -0.075*  [-0.122,-0.008] |  | -0.003  [-0.026,0.020] | -0.003  [-0.026,0.020] | -0.048*  [-0.095,-0.001] |

MM: multimorbidity; CI: confidence interval; MSK: musculoskeletal.

Models adjusted for number of chronic diseases, age, sex, education, Mini Mental State Examination, Body mass index, and number of medications.

Reference group: individuals without multimorbidity (*n***=**415) or individuals in MM pattern “Unspecific” (*n***=**1246)

95% confidence intervals in brackets;

* results statistically significant

Table S8. Associations between chronic diseases count and multimorbidity patterns and annual change (β coefficients) in walking speed test (z-score), chair stand test (z-score) and combined physical function measure (z-score) over the 15-year follow-up after excluding individuals who developed dementia within the first 6 years of follow-up.

|  |  |  | **MM Patterns** | | | | |
| --- | --- | --- | --- | --- | --- | --- | --- |
|  |  | **Unspecific**  **(***n***=985)** | | **Psychiatric, respiratory & MSK**  **(***n***=372)** | **Anemia & sensory impairment**  **(***n***=455)** | **Cardiometabolic & inflammatory**  **(***n***=272)** |  |
| **Combined measure**  **(z-score)** | |  | |  |  |  |  |
|  |  | -0.023*  [-0.036,-0.010] | | -0.018  [-0.038,0.001] | -0.038*  [-0.059,-0.017] | -0.066*  [-0.115,-0.018] |  |
| **Walking speed test**  **(z-score)** | |  | |  |  |  |  |
|  |  | -0.019*  [-0.031,-0.006] | | -0.015  [-0.033,0.003] | -0.035*  [-0.056,-0.015] | -0.051  [-0.105,0.002] |  |
| **Chair stand test**  **(z-score)** | |  | |  |  |  |  |
|  |  | -0.030*  [-0.044,-0.014] | | -0.025  [-0.051,0.000] | -0.039*  [-0.069,-0.009] | -0.078*  [-0.129,-0.028] |  |

MM: multimorbidity; CI: confidence interval; MSK: musculoskeletal.

Models adjusted for age, sex, education, Mini Mental State Examination, Body mass index, and number of medications.

Reference group: individuals without multimorbidity (*n***=**336)

95% confidence intervals in brackets;

* results statistically significant

Table S9. Follow-up of the study population by disease patterns (n=3112).

|  | **Total** | **No MM** | **Unspecific** | **Psychiatric, respiratory & MSK** | **Anemia & sensory impairment** | **Cardiometabolic & inflammatory** |
| --- | --- | --- | --- | --- | --- | --- |
|  | **N=3,112** | **N=421 (13.5%)** | **N=1,246 (40%)** | **N=482 (15.4%)** | **N=609 (19.6%)** | **N=354 (11.4%)** |
| Median follow-up duration, yr (IQR) | 6.7 (11.6) | 11.5 (5.9) | 10.9 (8.1) | 6.4 (9.4) | 5.5 (9.1) | 2.7 (5.8) |
| Complete 15-year follow-up, *n* (%)* | 916 (29.4%) | 247 (58.7%) | 446 (35.8%) | 138 (28.6%) | 73 (11.9%) | 12 (3.4%) |
| Complete 12-year follow-up, *n* (%)* | 1188 (38.1%) | 268 (63.6%) | 579 (46.5%) | 182 (37.8%) | 129 (21.2%) | 30 (8.4%) |
| Individuals that died during follow-up, *n* (%) | 1,405 (45.2%) | 73 (17.3%) | 445 (35.7%) | 201 (41.7%) | 395 (64.9%) | 291 (82.2%) |
| Individuals that drop during follow-up, *n* (%) | 791 (25.4%) | 101 (24.0%) | 355 (28.5%) | 143 (29.7%) | 141 (23.2%) | 51 (14.4%) |

IQR: interquartile range; MM: multimorbidity; MSK: musculoskeletal.

*We also report data from the 12-year follow-up, as the 15-year time point represents an intermediate assessment at which not all participants in the study population were evaluated, in accordance with the study design.

Table S10. Associations between multimorbidity patterns and annual change (β coefficients) in walking speed test (z-score), chair stand test (z-score) and combined physical function measure (z-score) over the 15-year follow-up (*n*=3036) with and without IPW.

|  |  | **With IPW** | | | |  | **Without IPW** | | | |
| --- | --- | --- | --- | --- | --- | --- | --- | --- | --- | --- |
|  |  | **Unspecific**  **(***n***=1231)** | **Psychiatric, respiratory & MSK**  **(***n***=470)** | **Anemia & sensory impairment**  **(***n***=587)** | **Cardiometabolic & inflammatory**  **(***n***=333)** |  | **Unspecific**  **(***n***=1231)** | **Psychiatric, respiratory & MSK**  **(***n***=470)** | **Anemia & sensory impairment**  **(***n***=587)** | **Cardiometabolic & inflammatory**  **(***n***=333)** |
| **Combined measure**  **(z-score)** | |  |  |  |  |  |  |  |  |  |
|  |  | -0.025  [-0.037,-0.012] | -0.024  [-0.042,-0.006] | -0.043  [-0.063,-0.023] | -0.066  [-0.111,-0.021] |  | -0.027  [-0.036,-0.016] | -0.028  [-0.040,-0.016] | -0.050  [-0.062,-0.038] | -0.056  [-0.073,-0.038] |
| **Walking speed test**  **(z-score)** | |  |  |  |  |  |  |  |  |  |
|  |  | -0.025  [-0.037,-0.012] | -0.024  [-0.042,-0.006] | -0.043  [-0.063,-0.023] | -0.066  [-0.111,-0.021] |  | -0.024  [-0.033,-0.014] | -0.028  [-0.040,-0.016] | -0.047  [-0.059,-0.035] | -0.037  [-0.055,-0.019] |
| **Chair stand test**  **(z-score)** | |  |  |  |  |  |  |  |  |  |
|  |  | -0.027  [-0.043,-0.012] | -0.030  [-0.054,-0.007] | -0.043  [-0.071,-0.015] | -0.075  [-0.123,-0.027] |  | -0.028  [-0.040,-0.015] | -0.027  [-0.042,-0.012] | -0.048  [-0.062,-0.032] | -0.066  [-0.088,-0.044] |

MM: multimorbidity; CI: confidence interval; MSK: musculoskeletal.

Models adjusted for age, sex, education, Mini Mental State Examination, Body mass index, and number of medications.

Reference group: individuals without multimorbidity (*n***=**415)

95% confidence intervals in brackets;
All results were statistically significant.

Table S11. Associations between multimorbidity patterns and annual change (β coefficients) in the combined physical function measure (z-score) over the 15-year follow-up (*n*=3036) with extra-adjustment for institutionalization.

|  |  |  | **With adjustment for institutionalization** | | | |
| --- | --- | --- | --- | --- | --- | --- |
|  |  |  | **Unspecific**  **(***n***=1231)** | **Psychiatric, respiratory & MSK**  **(***n***=470)** | **Anemia & sensory impairment**  **(***n***=587)** | **Cardiometabolic & inflammatory**  **(***n***=333)** |
| **Combined measure**  **(z-score)** | |  |  |  |  |  |
|  |  |  | -0.025  [-0.037,-0.012] | -0.024  [-0.042,-0.006] | -0.043  [-0.063,-0.023] | -0.066  [-0.111,-0.021] |

MM: multimorbidity; CI: confidence interval; MSK: musculoskeletal.

Models adjusted for age, sex, education, Mini Mental State Examination, Body mass index, and number of medications.

Reference group: individuals without multimorbidity (*n***=**415)

95% confidence intervals in brackets;
All results were statistically significant.

Table S12. Numbers of walking speed test values imputed due to inability to perform the test across multimorbidity patterns, by wave.

|  |  | |  | | | | |  | **MM Patterns** | | | | | | | | | |  |
| --- | --- | --- | --- | --- | --- | --- | --- | --- | --- | --- | --- | --- | --- | --- | --- | --- | --- | --- | --- |
|  |  | |  | | **Total** | |  | | | **No MM** | | **Unspecific** | | **Psychiatric, respiratory & MSK** | | **Anemia & sensory impairment** | | **Cardiometabolic & inflammatory** | |
| **Baseline** | |  | | 31 | |  | | | 0 (0%) | | 8 (25.8%) | | 6 (19.4%) | | 12 (38.7%) | | 5 (16.1%) | |  |
| **3-year follow-up** | |  | | 130 | |  | | | 2 (1.5%) | | 24 (18.5%) | | 21 (16.2%) | | 51 (39.2%) | | 32 (24.6%) | |  |
| **6-year follow-up** | |  | | 152 | |  | | | 7 (4.6%) | | 41 (26.9%) | | 31 (20.5%) | | 50 (32.9%) | | 23 (15.1%) | |  |
| **9-year follow-up** | |  | | 112 | |  | | | 4 (3.6%) | | 31 (27.9%) | | 26 (23.0%) | | 41 (36.6%) | | 10 (8.9%) | |  |
| **12-year follow-up** | |  | | 96 | |  | | | 3 (3.1%) | | 39 (40.6%) | | 21 (21.9%) | | 28 (29.2%) | | 5 (5.2%) | |  |
| **15-year follow-up** | |  | | 65 | |  | | | 5 (7.8%) | | 29 (44.6%) | | 14 (21.5%) | | 14 (21.5%) | | 3 (4.6%) | |  |

Numbers represent n (%). MM: multimorbidity; MSK: musculoskeletal.

Table S13. Distribution of chair stands test values imputed due to inability to perform the test across multimorbidity patterns, by wave.

|  |  |  | | |  | **MM Patterns** | | | | |  |
| --- | --- | --- | --- | --- | --- | --- | --- | --- | --- | --- | --- |
|  |  |  | **Total** |  | | **No MM** | **Unspecific** | **Psychiatric, respiratory & MSK** | **Anemia & sensory impairment** | **Cardiometabolic & inflammatory** | |
| **Baseline** | |  | 93 |  | | 6 (6.5) | 34 (36.6) | 23 (24.7) | 22 (23.6) | 8 (8.6) | |
| **3-year follow-up** | |  | 79 |  | | 4 (5.1) | 18 (22.8) | 9 (11.4) | 31 (39.2) | 17 (21.5) | |
| **6-year follow-up** | |  | 206 |  | | 21 (10.2) | 77 (37.4) | 38 (18.4) | 50 (24.3) | 20 (9.7) | |
| **9-year follow-up** | |  | 149 |  | | 11 (7.4) | 72 (48.3) | 18 (12.1) | 38 (25.5) | 10 (6.7) | |
| **12-year follow-up** | |  | 213 |  | | 32 (15.0) | 114 (53.5) | 31 (14.5) | 28 (13.2) | 8 (3.8) | |
| **15-year follow-up** | |  | 119 |  | | 29 (24.3) | 56 (47.1) | 20 (16.8) | 10 (8.4) | 4 (3.4) | |

MM: multimorbidity; MSK: musculoskeletal.

Table S14 . Associations between multimorbidity patterns and 15-year annual change in walking speed (m/s) and chair stand time (s), with inability to perform imputed using the 90^th^ and 75^th^ percentile values of the study population.

|  | | **MM Patterns** | | | | | | | | | | |
| --- | --- | --- | --- | --- | --- | --- | --- | --- | --- | --- | --- | --- |
|  |  | **Unspecific**  **(***n***=1231)** | |  | **Psychiatric, respiratory & MSK**  **(***n***=470)** | |  | **Anemia & sensory impairment**  **(***n***=587)** | |  | **Cardiometabolic & inflammatory**  **(***n***=333)** | |
|  |  | 90th | 75th |  | 90th | 75th |  | 90th | 75th |  | 90th | 75th |
| **Walking speed test**  **(m/s)** |  |  |  |  |  |  |  |  |  |  |  |  |
|  |  | -0.009*  [-0.012,-0.005] | -0.008*  [-0.012,-0.004] |  | -0.009*  [-0.014,-0.004] | -0.007*  [-0.011,-0.002] |  | -0.015*  [-0.019,-0.011] | -0.012*  [-0.017,-0.008] |  | -0.010*  [-0.017,-0.003] | -0.006  [-0.013,0.002] |
| **Chair stands test**  **(s)** |  |  |  |  |  |  |  |  |  |  |  |  |
|  |  | 0.315*  [0.183,0.447] | 0.291*  [0.154,0.426] |  | 0.377*  [0.21,0.541] | 0.367*  [0.198,0.536] |  | 0.481*  [0.311,0.650] | 0.422*  [0.247,0.596] |  | 0.692*  [0.437,0.946] | 0.593*  [0.331,0.855] |

The 90th and 75th percentile values for walking speed were 0.488 and 0.813, respectively; corresponding values for the chair stands test were 22 s and 16 s.

MM: multimorbidity; CI: confidence interval; MSK: musculoskeletal.

Models adjusted for age, sex, education, Mini Mental State Examination, Body mass index, and number of medications.

Reference group: individuals without multimorbidity (*n***=**415)

95% confidence intervals in brackets;

* results statistically significant

This table shows the results from fitting linear mixed effect models with longitudinal physical function test as outcomes and baseline multimorbidity patterns as exposures. β-coefficients are interpreted as 1) walking speed test decline/year per each multimorbidity pattern compared to the no multimorbidity group, and 2) chair stand test time increase/year per each multimorbidity pattern compared to the no multimorbidity group.

Table S15. STROBE statement checklist for cohort studies.

|  | **Item No** | **Recommendation** | **Section** |
| --- | --- | --- | --- |
| **Title and abstract** | 1 | (*a*) Indicate the study’s design with a commonly used term in the title or the abstract | Title |
|  |  | (*b*) Provide in the abstract an informative and balanced summary of what was done and what was found | Abstract |
| **Introduction** | | |  |
| Background/rationale | 2 | Explain the scientific background and rationale for the investigation being reported | Introduction |
| Objectives | 3 | State specific objectives, including any prespecified hypotheses | Introduction |
| **Methods** | | |  |
| Study design | 4 | Present key elements of study design early in the paper | Methods (1) |
| Setting | 5 | Describe the setting, locations, and relevant dates, including periods of recruitment, exposure, follow-up, and data collection | Methods (1) |
| Participants | 6 | (*a*) Give the eligibility criteria, and the sources and methods of selection of participants. Describe methods of follow-up | Methods (1) |
|  |  | (*b*) For matched studies, give matching criteria and number of exposed and unexposed | N/A |
| Variables | 7 | Clearly define all outcomes, exposures, predictors, potential confounders, and effect modifiers. Give diagnostic criteria, if applicable | Methods (2) |
| Data sources/ measurement | 8* | For each variable of interest, give sources of data and details of methods of assessment (measurement). Describe comparability of assessment methods if there is more than one group | Methods (3-6) |
| Bias | 9 | Describe any efforts to address potential sources of bias | Methods (3-6) |
| Study size | 10 | Explain how the study size was arrived at | Methods (3-6) |
| Quantitative variables | 11 | Explain how quantitative variables were handled in the analyses. If applicable, describe which groupings were chosen and why | Methods (7) |
| Statistical methods | 12 | (*a*) Describe all statistical methods, including those used to control for confounding | Methods (7) |
|  |  | (*b*) Describe any methods used to examine subgroups and interactions | Methods (7) |
|  |  | (*c*) Explain how missing data were addressed | Methods (7) |
|  |  | (*d*) If applicable, explain how loss to follow-up was addressed | Methods (7) |
|  |  | (*e*) Describe any sensitivity analyses | Methods (7) |
| **Results** | | |  |
| Participants | 13* | (a) Report numbers of individuals at each stage of study—eg numbers potentially eligible, examined for eligibility, confirmed eligible, included in the study, completing follow-up, and analysed | Results |
|  |  | (b) Give reasons for non-participation at each stage | Appendix |
|  |  | (c) Consider use of a flow diagram | Appendix |
| Descriptive data | 14* | (a) Give characteristics of study participants (eg demographic, clinical, social) and information on exposures and potential confounders | Results |
|  |  | (b) Indicate number of participants with missing data for each variable of interest | Results |
|  |  | (c) Summarise follow-up time (eg, average and total amount) | Results |
| Outcome data | 15* | Report numbers of outcome events or summary measures over time | Results |
| Main results | 16 | (*a*) Give unadjusted estimates and, if applicable, confounder-adjusted estimates and their precision (eg, 95% confidence interval). Make clear which confounders were adjusted for and why they were included | Results |
|  |  | (*b*) Report category boundaries when continuous variables were categorized | Results |
|  |  | (*c*) If relevant, consider translating estimates of relative risk into absolute risk for a meaningful time period | N/A |
| Other analyses | 17 | Report other analyses done—eg analyses of subgroups and interactions, and sensitivity analyses | Results/Appendix |
| **Discussion** | | |  |
| Key results | 18 | Summarise key results with reference to study objectives | Discussion |
| Limitations | 19 | Discuss limitations of the study, taking into account sources of potential bias or imprecision. Discuss both direction and magnitude of any potential bias | Discussion |
| Interpretation | 20 | Give a cautious overall interpretation of results considering objectives, limitations, multiplicity of analyses, results from similar studies, and other relevant evidence | Discussion |
| Generalisability | 21 | Discuss the generalisability (external validity) of the study results | Discussion |
| **Other information** | | |  |
| Funding | 22 | Give the source of funding and the role of the funders for the present study and, if applicable, for the original study on which the present article is based | Other |
